# Living on the Edge: Emotional and Behavioral Problems in a Sample of Kosovar Veterans and Wives of Veterans 16 Years Postwar

**DOI:** 10.3389/fpsyt.2019.00598

**Published:** 2019-09-13

**Authors:** Mimoza Shahini, Leslie A. Rescorla, Merita Shala, Shqipe Ukshini

**Affiliations:** ^1^Department of Psychiatry, University Clinical Center of Kosovo, Pristina, Kosovo; ^2^Department of Psychology, Bryn Mawr College, Bryn Mawr, PA, United States; ^3^Department of Education, Mitrovica University, Mitrovica, Kosovo; ^4^Department of Psychology, University Clinical Center of Kosovo, Pristina, Kosovo

**Keywords:** war traumatic experience, emotional and behavioral problems, veterans, wives of veterans, predictor

## Abstract

**Aim:** This study aimed to explore the effects of war traumatic exposure on emotional and behavioral problems in a sample of Kosovar war veterans and the wives of veterans 16 years after the 1998–1999 war, as well as whether the level of education, income, well-being, and substance use are predictors for emotional and behavioral problems.

**Methods:** Self-report data were obtained from 373 adults, 247 male war veterans (66.2% of the sample) and 126 wives of other male war veterans (33.8% of the sample). The sample was recruited from a list of war veterans provided by the Kosovar National Association of War Veterans. The mean age of participants was 45.42 [standard deviation (SD), 7.64] years. Measurements comprised a sociodemographic brief structured interview, the Well-Being Index (WHO-5), the Harvard Trauma Questionnaire, and the Adult Self Report (ASR). Logistic regression analysis was conducted to explore if the demographic variables were predictors for ASR general scales and subscales. Multivariate analysis of covariance was performed by adding as covariates the continuous variables pointed out in the logistic regression analysis as discriminating factors between the groups. *Post hoc* analyses were corrected, and we estimated partial η^2^ to measure the effect size.

**Results:** The higher traumatic exposure during the war, the greater the tendency to have emotional problems and behavioral problems for both kinds of participants. The result showed that there were no differences on the prevalence of emotional and behavioral problems between the two groups, and both veterans and wives of veterans had no differences on seeking professional help for their emotional and behavioral problems. Wives of veterans living in rural areas showed higher scores on almost all ASR scales compared with those living in urban areas or even with those of veterans from urban and rural areas. Veterans with elementary education level had the highest scores compared with other groups. Veterans with poor well-being had the highest scores compared with other groups. Using Internalizing, Externalizing, and Total Problems as outcome variables and trauma exposure, smoking, drinking alcohol, and well-being as predictors, we found that the model was a significant predictor for both male and female participants on these three scales.

**Conclusion:** The relationship found between the level of exposure to traumatic events and emotional and behavior problems, as well as the factors that moderated such relations, in war veterans and their wives, should help global mental health researchers address the contextual dimensions of this relationship and identify better ways to prevent and treat those problems.

## Introduction

War and its consequences remain a challenge for mental health professionals because of specific dynamics that accompany individuals who were either witnesses and/or active participants in it. Studies have shown that conflict situations cause more mortality and disability than any major disease, destroying communities and families and often disrupting the social and economic development of nations (1). Nevertheless, the world is increasingly engaging in armed conflict, a significant part of which is not carried out by individuals trained in war craft, making their reactions even more complex. The war of 1998–1999 in Kosovo involved 36,000 people in combat, most of whom were not trained as professional soldiers.

A 2016 study in Kosovo (2) found a posttraumatic stress disorder (PTSD) prevalence of 11.2% in Kosovar war veterans 8 years after the conflict. Another study (3), which investigated long-term mental health outcome in Kosovo 8 years after the Balkans war, found a prevalence of 33% for PTSD or major depressive episode in a community sample. A large epidemiological survey conducted in the Balkans found prevalence for mood and anxious/depressed disorders of 47.6% and 41.8%, respectively, in 648 Kosovar adults (4).

Although many studies have studied combatants upon their return from service or combat, health professionals continue to debate about the effectiveness of the treatment of those who are faced with mental health problems, as well as about the impact of such problems on family members. A very important argument that should be considered in the context of this relationship is that the mental health problems of veterans are closely related not only to their service or participation in war and their traumatic exposure in the war, but also to the conditions in their lives when they return from war, how they perceive their situation, and the strategies they use to deal with stress. It is understandable that war causes deep moral dilemmas for every individual, but long-term consequences are related to the dilemma of survival, especially when their expectations are not met for the individuals, their families, and their society. Previous wars have demonstrated that veterans’ needs peak several decades after their war service, highlighting the necessity of managing current problems and planning for future needs (5).

During the 1998–1999 war, Kosovars were exposed to intense social disruptions including death of family members and being forcefully separated from home and family. People’s need for social support intensified at the same time that their support system was disrupted. The presence of psychiatric disorders and chronic use of experiential avoidance were expected to disrupt the process of trauma recovery and increase the difficulty of building the elements linked to high quality of life (6). Financial support, physical well-being, and other demographic variables remain to be explored in their relation with traumatic exposure and emotional and behavioral problems. This very complex context also includes the family, particularly the veterans’ wives.

The number of studies examining the effects of war on veterans continues to grow. However, the same cannot be said about research carried out on the effects of war wounds on the wives of veterans. The results are often focused on negative aspects, showing an increase in psychological problems among veterans’ wives (7, 8), with fewer studies focused on positive outcomes.

Posttraumatic stress disorder, the most common and perhaps the most visible result of participating in war, is characterized by a variety of symptoms of hypervigilance (9) with aggressive behavior (10, 11), as well as nonphysical forms of aggression (12). Posttraumatic stress disorder is often accompanied by an increased level of anxiety, depression (13), somatic symptoms, and difficulties in functioning.

In previous research, more than a third of war veterans’ wives were found to meet the criteria for secondary traumatic stress (14), and veterans’ PTSD was related to lower levels of marital adjustment (15–17). The findings of a considerable number of studies indicate that wives of veterans with PTSD are at an increasing risk of experiencing psychological problems and a lower level of marital adjustment than the general population (18–20). Furthermore, levels of avoidance, emotional numbness, and anger among veterans with PTSD are particularly connected with increased psychological and marital distress of their spouses (19).

The present study tested two hypotheses. The first hypothesis proposed that wartime traumatic exposure would predict emotional and behavioral problems in a sample of war veterans and the wives of veterans as assessed with Adult Self-Report (ASR). Building on this model, the second hypothesis stated that low level of education and income, poor well-being, and substance abuse would be additional predictors for a high level of emotional and behavioral problems.

## Methods

The study reported here is related to a larger study (unpublished manuscript, 2019) of the transfer of trauma through generations, which involves children of veterans of the 1998–1999 war in Kosovo. The war was fought by the Kosovo Liberation Army (KLA), which had been created to respond to Serbian repression against the Albanian population such as the massacre of 53 members of one family (21). Most people in the KLA did not have military training, and some of them did not even have any prior experience using weapons. There are no final data about the exact number of war veterans in Kosovo, as different sources present different numbers.

## Participants

We obtained self-report data from 373 adults, 247 male war veterans (66.2% of the sample) and 126 wives of other male war veterans (33.8% of the sample), a significant gender difference, *t*(373) = 39.25, *p* = 0.001 (**Table 1**). The sample was recruited from a list of 24,577 war veterans provided by the Kosovar National Association of War Veterans. This list, which was obtained from an Institute of Medicine (IOM) report, was compiled immediately after the war. However, it should be noted that there are various lists of Kosovar war veterans, with numbers given ranging from 13,000 to 47,000. The latter number most likely includes people who contributed to the war in noncombat roles (e.g., medical care, food supply, armaments supply, etc.) who registered on the longer list to obtain government benefits for war veterans. We used the IOM list as it seemed to be the most reliable source for actual KLA combat veterans. From this list, we identified 800 veterans from six regions of Kosovo who had at least one child of 6 to 18 years. We invited these 800 veterans to consent to their child’s participation in a study of the effects of the Kosovar war on children of veterans, and 574 veterans (71.8%) gave consent. On the day of the scheduled data collection interview, 247 veterans and 126 wives of other veterans came to the data collection site and completed the forms for their children, as well as three additional forms about themselves. Data for the remaining children were obtained from other family members (e.g., older brother/sisters or grandparents), who were not asked to complete the additional self-report measures.

**Table 1:** Distributions of demographic variables for participants.

Variables	Categories	N	%	Chi square
Subject Gender	Male	247	66.20%	χ^2^(1) = 39,25 p = 0.001
	Female	126	33.80%	
Ag groups	18- 35	31	8.30%	χ^2^(1) = 259.30 p = 0.001
	36-60	342	91.70%	
Place	urban	193	52.00%	χ^2^(1) = .606 p = 0.436
	rural	178	48.00%	
Education	Elementary	126	33.80%	
	Secondary	143	38.30%	χ^2^(2) = 6.15 p = 0.046
	Higher	104	27.90%	
Do you smoke?	Yes	153	41.00%	
	Sometimes	25	6.70%	χ^2^(2) = 126.13 p = 0.001
	No	195	52.30%	
Do you drink alcohol	Yes	10	2,70%	
	Sometimes	60	16.10%	χ^2^(2) = 395.16 p =0.001
	No	303	81.20%	
Do you have any illness	No	301	80.70%	χ^2^(1) = 140.59 p = 0.001
	Yes	72	19.30%	
Have you ever consulted a health professionals’	No	334	89.50%	χ^2^(1) = 233.31 p = 0.01
	Yes	39	10.50%	
	Very low	91	24.4%	
Incomes	Low	189	50.4%	
	Moderate	81	21.7%	χ^2^(3) = 167.1 p = 0.001
	High	13	3.5%	
Wellbeing	Poor wellbeing	84	22.5%	
	Moderate	184	49.3%	χ^2^(3) = 44.7 p = 0.001
	Better wellbeing	105	28.2%	
Traumatic experience during the war	High	16	4.40%	
	Moderate	185	50.50%	χ^2^(2) = 139.78 p = 0.001
	Low	165	45.10%	

The mean age of participants was 45.42 (SD, 7.64) years, which did not differ significantly by gender (*t*(371) = 1.64, *p* = 0.101). Education level of participants was 33.8% (n = 126) with elementary education, 38.3% (n = 143) with secondary education, and 27.9% (n = 104) with higher education. There was no association between gender and education level (χ^2^(2) = 1.68, *p* < 0.431). Incomes were categorized according to average public sector income per month in Kosova: 0 to 200 euros (very low income; people with social assistance) = 24% (n = 91); 201 to 400 euros (low income) = 50.4% (n = 188); 401 to 800 euros (moderate income) = 21.7% (n = 81); and greater than 800 euros (high income) = 3.5% (n = 13). There was no association between gender and income (χ^2^(3) = 1.12, *p* < 0.771).

## Measures

Sociodemographic sample characteristics were assessed using a brief structured interview. Wartime exposure and symptom severity on emotional and behavioral problems were assessed by means of the following three self-report questionnaires.

**Well-Being Index (WHO-5)**. The WHO-5 is among the most widely used questionnaires assessing subjective psychological well-being. The WHO-5 is a short questionnaire consisting of five simple and noninvasive questions that tap into the subjective well-being of the respondents. This questionnaire has been translated in 30 languages and has been used in research studies all over the world. Our sample also showed very good internal consistency, with Cronbach’s α = 0.891 for the total sample, veterans α = 0.903 and wives α = 0.859. Higher scores mean better well-being.

**Harvard Trauma Questionnaire**. Part 1 (The Trauma Events Scale) of the Harvard Trauma Questionnaire (HTQ-R) assesses 41 categories of traumatic life events that respondents may have experienced during the war (e.g., as “combat situation” and “forced separation from family members”), with response options of “yes” and “no.” The HTQ has demonstrated good internal consistency (22, 23) across culturally distinct populations. Our sample also showed very good internal consistency on the HTQ-R, with Cronbach’s α = 0.856 for the total sample, veterans α = 0.858 and wives α = 0.853. From the sum of all variables in this questionnaire, we created a new variable in distinct categories using a cutoff of +2 SDs. The categories of traumatic exposure were 1 = low exposed group = 1 SD, 2 = moderate exposed group >1 SD − <2 SD, 3 = high exposed group >2 SDs above mean, level of exposure during the war.

**Adult Self-Report Form**. The ASR questionnaire (24) asks participants questions about what they have experienced in the last 6 months. The ASR has 134 items, 11 of which describe socially desirable qualities and the rest of which describe various behavioral, emotional, social, and thought problems. Completion time is generally between 25 and 40 min. For most responses, participants use a three-level Likert scale: 0 = not true, 1 = somewhat or sometimes true, 2 = very true or often true. The last three items consist of questions about the number of days in the past 6 months the respondent has used tobacco, alcohol, and drugs.

The ASR has eight empirically based syndrome scales Anxious/Depressed: Anxiety-Depression, Withdrawn, Somatic Complaints, Thought Problems, Attention Problems, Aggressive Behavior, Rule-Breaking Behavior, and Intrusive Behavior. The sum of the scores on the Anxiety-Depression, Withdrawn, and Somatic Complaints scales yields a broad-spectrum Internalizing Problems score, whereas the sum of the Aggressive Behavior, Rule-Breaking Behavior, and Intrusive Behavior scores yields a broad-spectrum Externalizing score. The third broad-spectrum scale is Total Problems, the sum of all problem items on the form. The ASR also has six *Diagnostic and Statistical Manual of Mental Disorders* (DSM)–oriented scales: Depressive Problems, Anxiety Problems, Somatic Problems, Avoidant Personality Problems, Attention-Deficit Hyperactivity (ADH) Problems (with Inattention and Hyperactivity-Impulsivity subscales), and Antisocial Personality Problems, plus two scales based on research conducted by others (Obsessive-Compulsive Problems and Sluggish Cognitive Tempo). For all problem scales, a higher score represents a higher severity. The 11 personal strengths items (e.g., “I make good use of my opportunities”) are summed to yield a Personal Strengths scale score, with higher scores indicating more strengths. In our sample, the ASR manifested excellent internal consistency: Cronbach’s α = 0.942 for the total sample, α = 0.941 for veterans and α = 0.943 for wives. There were no significant differences in α by gender.

## Statistical Analyses

The main descriptive data (mean, SD, frequencies) were calculated for all variables. Differences in sociodemographic and clinical variables were investigated with χ^2^ or analyses of variance (ANOVAs) for categorical and continuous variables, respectively.

*T* scores for all ASR scales were calculated based in Kosovo population norms (25). Logistic regression analysis was conducted to explore the possible prediction of demographic variables on ASR broad scales and subscales. Statistical performance of *R* ^2^ effect-size measures (26) also provided an effect size estimate of the variance accounted for by the indirect effect.

Multivariate analysis of covariance analysis was performed by adding as covariates the continuous variables identified in the logistic regression analysis as discriminating factors between the groups. *Post hoc* analyses were corrected, and we estimated partial η^2^ to measure the effect size. All data were analyzed using SPSS/PC software version 24.0, and all statistical tests were bilateral with a *p* ≤ 0.01.

## Results

We defined the prevalence of significant emotional and behavioral problems using a *T* score cutoff point of 60 (84th percentile, borderline + clinical range). For veterans and for wives of veterans, respectively, prevalence was 8.1% and 11.9% for Total Problem scores, 10.1% and 11.1% for Internalizing Problems, and 6.9% and 7.1% for Externalizing. There were no gender differences on the prevalence rate for these broad-spectrum ASR scales.

## Effects of Demographic Factors on ASR Scores

**Gender.** Means and SDs for male veterans and wives of veterans on all ASR problem scales are presented in **Table 2**, with gender differences tested using independent-sample *t* tests. Females had higher scores than males on only one of the ASR scale, namely, Somatic Complaints, *t* = −2.48, *degrees of freedom* (*df*) = 371, *p* = 0.014. No significant differences were found based on religion or place of residence (rural vs. urban).

**Table 2:** Prevalence of empirical Total Problems of ASR according to gender.

		Male	Female
	Normal	95.1%	96%
Anxious/Depressed	Borderline	2%	2.4%
	Clinical	2.8%	1.6%
	Normal	92.7%	90.5%
Withdrawn	Borderline	3.6%	5.6%
	Clinical	3.6%	4%
	Normal	86.2%	88.9%
Somatic Complaints	Borderline	7.3%	6.3%
	Clinical	6.5%	4.8%
	Normal	78.9%	81%
Thought Problems	Borderline	12.6%	11.1%
	Clinical	8.5%	7.9%
	Normal	93.9%	93.7%
Attention Problems	Borderline	3.6%	4.8%
	Clinical	2.4%	1.6%
	Normal	93.9%	94.4%
Aggressive Behavior	Borderline	3.2%	4%
	Clinical	2.8%	1.6%
	Normal	92.7%	92.1%
Rule-breaking Behavior	Borderline	4.9%	4.8%
	Clinical	2.4%	3.2%
	Normal	91.1%	88.9%
Intrusive	Borderline	7.7%	7.1%
	Clinical	1.2%	4%
	Normal	82.2%	83.3%
Internalizing	Borderline	7.7%	5.6%
	Clinical	10.1%	11.1%
	Normal	85%	81%
Externalizing	Borderline	8.1%	11.9%
	Clinical	6.9%	7.1%
	Normal	82.2%	79.4%
Total	Borderline	9.7%	8.7%
	Clinical	8.1%	11.9%
	Low strengths	87.4%	89.7%
Personal Strengths	Moderate strengths	3.6%	1.6%
	Higher strengths	8.5%	8.7%

**Residence.** Although no main effect of place of living was found, visual inspection of mean scores suggested a possible gender × residence interaction, which was tested in a series of two-way ANOVAs (**Table 3**). Significant interactions were found only for Internalizing Problems, *F* = 6.60, *df* = 1, *p* = 0.01); η^2^ = 0.02. As seen in **Table 2**, females living in rural areas had higher scores on these scales than those living in urban areas and then males in either urban or rural areas.

**Table 3:** Mean and standard deviation for ASR Total Problems and t test results according to gender and place of living.

	Male	Female	Urban	Rural
	Mean (SD)	Mean (SD)	Mean (SD)	Mean (SD)
Anxious/Depressed	6.1(4.9)	7.1(4.9)	6.8(5.3)	5.91(4.3)
Withdrawn	2.8(2.7)	2.8(2.7)	2.9(2.9)	2.7(2.4)
Somatic Complaints	3.5(3.7)	4.5(3.9)	4.1(4.1)	3.5(3.4)
Thought Problems	2.8(2.5)	3.1(3.2)	3.1(2.6)	2.7(2.9)
Attention Problems	4.3(4.2)	5.3(4.1)	4.8(4.6)	4.4(3.6)
Aggressive Behavior	4.2(3.8)	4.3(3.4)	4.4(4.1)	4.1(3.1)
Rule-breaking Behavior	2.5(2.8)	2.1(2.6)	2.5(2.5)	2.1(2.5)
Intrusive	2.8(2.1)	2.8(2.1)	2.9(2.1)	2.6(1.8)
Internalizing problems	12.4(9.7)	14.4(9.9)	13.8(10.7)	12.2(8.5)
Externalizing problems	9.6(7.3)	9.2(6.6)	10.1(7.8)	8.8(6.1)
Total of emotional and behavioral problems	37.7(26.1)	41.2(24.1)	40.7(28.1)	36.4(22.2)
Personal strength	16.1(5.9)	15.6(4.5)	16.1(4.3)	15.6(6.4)

**Income.** We also analyzed the effects of income on ASR scores. For males, income category had a significant effect on Anxious/Depressed, *F* = 3.96, *df* = 3, *p* = 0.009, η^2^ = 0.047; Withdrawn *F* = 3.72, *df* = 3, *p* = 0.01, η^2^ = 0.04; and Attention Problems *F* = 3.76, *df* = 3, *p* = 0.01, η^2^ = 0.04. *Post hoc* tests indicated that the only significant pairwise effects (*p* < 0.01) were between the very low-income group and the moderate-income group. Male participants from the very low-income group had significanly higher scores on *DSM*–Depressive Problems, *DSM*–Avoidant Personality Problems, and *DSM*-Inattention, compared with those with low and moderate income.

For female participants, income category had a significant effect on Anxious/Depressed, *F* = 4.57, *df* = 3, *p* = 0.005, η^2^ = 0.101, and Internalizing Problems scores, *F* = 4.06, *df* = 3, *p* < 0.009, η^2^ = 0.09. *Post hoc* tests indicated significant differences between the very low-income group and both the low- and moderate-income groups (*p* < 0.01). For female participants, significant differences were also found in *DSM*–Depressive Problems; *DSM*-Anxiety Problems; *DSM*–Avoidant Personality Problems, and *DSM*-Inattention.

**Education.** Participants were categorized into three groups based on education: elementary (E), secondary (S), and higher education (H). Significant effects for education level were found for male participants on Total Problems, *F* = 4.44, *df* = 2, *p* = 0.01, η^2^ = 0.04. According to Bonferroni correction tests, the elementary education group had significantly higher scores than those in the 2 higher education groups. When multivariate analyses of variance were conducted to test the effect of education level on the ASR subscales, results were significant for four scales for males, with the lowest problem scores in the higher education group and the highest problem scores in the elementary education group: Attention Problems, *F* = 7.32, *df* = 2, *p* = 0.001, η^2^ = 0.058 (H < S < E); and Rule-Breaking Behavior, *F* = 7.30, *df* = 2, *p* = 0.001, η^2^ = 0.06 (H < S < E). Male participants with higher education were also found to have significantly lower scores compared with the elementary education group on *DSM*–Depressive Problems; *DSM*–Avoidant Personality Problems, *DSM*-Inattention, and *DSM*–Antisocial Personality Problems.

For female participants, education level did not have a significant effect on the ASR broad scales. Education categories for female participants were found to have significant effects only in *DSM*-Inattention, with the elementary education group having significantly higher scores compared with those with higher education, but not to those with secondary education.

**Traumatic Exposure Effects.** As described above, traumatic exposure was categorized as low (L), moderate (M), and high (H) for both male and female participants. Significant effects for traumatic exposure were found for male participants on most of scales: Anxious/Depressed, *F* = 10.3, *df* = 2, *p* = 0.001, η^2^ = 0.024; Withdrawn, *F* = 7.8, *df* = 2, *p* = 0.001, η^2^ = 0.03; Attention Problems, *F* = 11.1, *df* = 2, *p* = 0.001, η^2^ = 0.058; Aggressive Behavior, *F* = 11.9, *df* = 2, *p* = 0.001, η^2^ = 0.01; Rule-Breaking Behavior, *F* = 11.6, *df* = 2, *p* = 0.001, η^2^ = 0.06; Internalizing, *F* = 11.5, *df* = 2, *p* = 0.01, η^2^ = 0.03; Externalizing, *F* = 11.1, *df* = 2, *p* = 0.01, η^2^ = 0.03; and Total Problems, *F* = 12.3, *df* = 2, *p* = 0.01, η^2^ = 0.04 (**Table 4**). According to *post hoc* analysis, male participants with low traumatic exposure during the war reported lower scores than males with moderate or high traumatic exposure during the war (*p* < 0.01). On the other hand, no significant differences for traumatic exposure were found for males on the Intrusive syndrome or the Personal Strengths scale. Traumatic exposures during the war was found to have a significant effect on the mean scores of all *DSM* scales for male participants, with participants with higher exposure having higher scores on all *DSM* scales (*p* < 0.01) compared with those with low and moderate exposure during the war.

**Table 4:** ANOVA results for Total Problems of ASR according to traumatic experience, SES, education, drinking and smoking.

	Trauma exposure		SES		Education		Drinking		Smoking	
	F	p	F	p	F	p	F	p	F	p
Anxious/Depressed	12.91	0.001	3.71	0.012	4.80	0.009	9.99	0.001	7.03	0.001
Withdrawn	8.40	0.001	1.59	NS	3.66	0.026	4.91	0.008	4.82	0.009
Somatic Complaints	10.57	0.001	0.638	NS	3.54	0.030	1.70	NS	2.62	NS
Thought Problems	2.89	NS	1.53	NS	0.436	NS	10.50	0.001	3.56	0.029
Attention Problems	15.22	0.001	2.29	NS	11.29	0.001	6.94	0.001	4.66	0.010
Aggressive Behavior	13.86	0.001	3.28	0.021	3.46	0.032	7.48	0.001	7.05	0.001
Rule-breaking Behavior	12.35	0.001	7.75	0.001	6.61	0.002	2.65	NS	4.89	0.008
Intrusive	1.39	NS	0.202	NS	1.07	NS	2.49	NS	4.64	0.010
Internalizing problems	15.22	0.001	5.15	0.002	5.59	0.004	7.24	0.001	6.60	0.002
Externalizing problems	13.44	0.001	2.11	NS	3.72	0.025	5.86	0.003	8.37	0.001
Total of emotional and behavioral problems	15.25	0.001	3.53	0.015	5.24	0.006	8.41	0.001	7.61	0.001
DSM Scales										
Depressive Problems	14.21	0.001	5.75	0.001	7.73	0.001	3.08	0.04	5051	0.004
Anxiety Problems	9.70	0.001	2.18	NS	3.28	0.04	2.12	NS	3.07	0.047
Somatic Problems	8.62	0.001	1.56	NS	3.37	0.03	.767	NS	2.83	NS
Avoidant Personality Problems	11.11	0.001	4.67	0.003	4.68	0.01	4.47	0.01	4.48	0.01
Attention Deficit Hyperactivity (ADH) Problems	15.32	0.001	1.98	NS	3.77	0.02	8.60	0.001	5.90	0.003
Inattention subscale	15.66	0.001	4.69	0.003	9.48	0.001	6.76	0.001	5.13	0.006
Hyperactivity-Impulsivity subscale	8.88	0.001	.263	NS	.370	NS	6.76	0.001	4.50	0.012
Antisocial Personality Problems	13.91	0.001	2.57	0.05	7.50	0.001	2.26	NS	5.18	0.006
Obsessive-Compulsive Problems	2.63	NS	.132	NS	.358	NS	7.83	0.001	4.32	0.014

For females, traumatic exposure had significant effects on Somatic Complaints, *F* = 7.12, *df* = 2, *p* = 0.01, η^2^ = 0.11 (H > L > M); Attention Problems, *F* = 4.51, *df* = 2, *p* = 0.01, η^2^ = 0.07 (H > M > L); and Internalizing Problems, *F* = 4.61, *df* = 2, *p* = 0.01, η^2^ = 0.07 (M > H > L). For female participants, traumatic exposure was found to have significant effect on Depressive Problems; Somatic Problems; ADHP; and Inattention. Females with higher traumatic exposure had significant differences compared with those with a low level of exposure.

**Well-being**. Participants were categorized into three groups based on well-being in poor (P), moderate (M), and better (B). Significant effects for well-being were found for male participants on 11 scales: Anxious/Depressed, *F* = 6.82, *df* = 2, *p* = 0.001, η^2^ = 0.05; Withdrawn, *F* = 7.82, *df* = 2, *p* = 0.001, η^2^ = 0.06; Somatic Complaints, *F* = 5.00, *df* = 2, *p* = 0.007, η^2^ = 0.04; Thought Problems, *F* = 4.52, *df* = 2, *p* = 0.012, η^2^ = 0.04; Attention Problems (*F* = 12.28, *df* = 2, *p* = 0.000, η^2^ = 0.09; Aggressive Behavior, *F* = 5.14, *df* = 2, *p* = 0.007, η^2^ = 0.04; Rule-Breaking Behavior, *F* = 6.99 *df* = 2, *p* = 0.001, η^2^ = 0.05; Internalizing, *F* = 8.66, *df* = 2, *p* = 0.00, η^2^ = 0.07; Externalizing, *F* = 5.22, *df* = 2, *p* = 0.006, η^2^ = 0.04; and Total Problems, *F* = 7.98, *df* = 2, *p* = 0.00, η^2^ = 0.046. According to Bonferroni correction tests, the better well-being group had significantly lower scores than those in the two other groups. Male participants with better well-being were also found to have significantly lower scores compared with the other group on *DSM*–Depressive Problems; *DSM*–Avoidant Personality Problems, *DSM*-ADHD; *DSM*-Inattention, and *DSM*–Antisocial Personality Problems (*p* < 0.01).

For females, well-being had significant effects on Anxious/Depressed, *F* = 10.50, *df* = 2, *p* = 0.000, η^2^ = 0.15; Withdrawn, *F* = 7.46, *df* = 2, *p* = 0.001, η^2^ = 0.11; Somatic Complaints, *F* = 4.76, *df* = 2, *p* = 0.01, η^2^ = 0.07; Attention Problems, *F* = 10.08, *df* = 2, *p* = 0.00, η^2^ = 0.14; Aggressive Behavior, *F* = 6.99, *df* = 2, *p* = 0.001, η^2^ = 0.10; Rule-Breaking Behavior, *F* = 6.00, *df* = 2, *p* = 0.003, η^2^ = 0.09; Internalizing Problems, *F* = 10.44, *df* = 2, *p* = 0.00, η^2^ = 0.15; Externalizing Problems, *F* = 4.51, *df* = 2, *p* = 0.01, η^2^ = 0.07; and Total Problems, *F* = 9.99, *df* = 2, *p* = 0.00, η^2^ = 0.14.

For female participants, well-being was found to have significant effect all *DSM*-scales, except for *DSM*–Obsessive-compulsive disorder (*p* > 0.01).

**Effects of Drinking and Smoking.** Drinking was categorized into the categories of regular drinker (R), nonregular drinker (NR), and nondrinker (N). Significant effects of drinking category were found for men on Anxious/Depressed, *F* = 5.09, *df* = 2, *p* = 0.007, η^2^ = 0.04 (R > NR > N); Thought Problems, *F* = 11,79, *df* = 2, *p* = 0.001, η^2^ = 0.088 (R > NR > N); Aggressive Behavior, *F* = 4.78, *df* = 2, *p* = 0.009, η^2^ = 0.038 (R > NR > N); and Total mean scores, *F* = 4.59, *df* = 2, *p* = 0.01, η^2^ = 0.036 (R > NR > N). Results of Bonferroni correction showed that regular drinkers had significantly higher mean scores than nondrinkers, and also nonregular drinkers had significant differences from nondrinkers. For *DSM* scales, drinking categories had a significant effect for male participants only on *DSM*–Avoidant Personality Problems, *DSM*-ADHP (and the Hyperactivity-Impulsivity subscale), and Obsessive-Compulsive Problems. Results showed that nonregular drinkers had significantly higher scores than nondrinkers on those four scales.

For females, drinking alcohol had a significant effect on Anxious/Depressed, *F* = 4.63, *df* = 2, *p* = 0.01, η^2^ = 0.07 (NR > R > N); Attention Problems, *F* = 4.37, *df* = 2, *p* = 0.01, η^2^ = 0.06 (R > NR > N); and Externalizing Problems, *F* = 4.07, *df* = 2, *p* = 0.01, η^2^ = 0.06 (NR > R > N). Significant differences according to Bonferroni correction were found between NR drinkers compared with nondrinkers and not found with regular drinkers. For female participants, drinking was related to only two *DSM* scales, ADHP and the Inattention subscale, with nonregular drinkers having higher scores that nondrinkers.

Multivariate analyses of variance indicated that smoking category (regular smokers = R, nonregular smokers = NR, and nonsmokers = N) had no significant effects on mean scores of any ASR scales for male participants, but it was significant for female participants on nine scales: Anxious/Depressed, *F* = 8.85, *df* = 2, *p* = 0.001, η^2^ = 0.126; Withdrawn, *F* = 7.63, *df* = 2, *p* = 0.001, η^2^ = 0.11; Somatic Complaints, *F* = 4.20, *df* = 2, *p* = 0.01, η^2^ = 0.06; Thought Problems, *F* = 4.52, *df* = 2, *p* = 0.01, η^2^ = 0.069; Attention Problems (*F* = 7.17, *df* = 2, *p* = 0.001, η^2^ = 0.11; Aggressive Behavior, *F* = 12.89, *df* = 2, *p* = 0.001, η^2^ = 0.17; Rule-Breaking Behavior, *F* = 8.25, *df* = 2, *p* = 0.001, η^2^ = 0.118; Intrusive, *F* = 4.73, *df* = 2, *p* = 0.01, η^2^ = 0.07 (NR vs. N, *p* = 0.02); and Total Problems, *F* = 12.69, *df* = 2, *p* = 0.001, η^2^ = 0.17. Female nonsmokers had lower scores in all ASR scales than the R and NR smoking groups (regular and nonregular group), but Bonferroni correction showed that only the differences between NR smokers and N smokers on Withdrawn, Somatic Complaints, Thought Problems, Attention Problems, and Intrusive (*p* < 0.01) were significant. Female participants from the category of nonregular smokers had significantly higher scores on all *DSM* scales (except Anxiety scale *p* > 0.01) compared with those in the nonsmokers group. Bonferoni correction showed that significant differences were found between nonsmokers with nonregular smokers in *DSM*–Somatic Problems, *DSM*–Avoidant Personality Problems, *DSM*-Inattention, and *DSM*–Antisocial Personality Problems. Between nonsmokers and regular smokers, significant differences were found in *DSM*–Depressive Problems and *DSM*-ADH Problems (and the Hyperactivity-Impulsivity).

A MANOVA was conducted to test the effect of Personal Strengths categories (low [L], moderate [M], higher strengths [H]) on ASR scales. Results showed that Personal Strengths categories had significant effects on almost all scales of ASR except, Somatic Complaints, Attention Problems, and Rule-Breaking Behavior for male participants. Male participants from the group with low personal strengths had significantly higher problem scores compared with participants from higher personal strengths on scales Withdrawn, *F* = 3.41, *df* = 2, *p* = 0.001, η^2^ = 0.055 (L > M > H); Intrusive, *F* = 6.76, *df* = 2, *p* = 0.001, η^2^ = 0.054 (L > M > H); Internalizing, *F* = 4.86, *df* = 2, *p* = 0.009, η^2^ = 0.038 (L > M > H); Externalizing, *F* = 3.83, *df* = 2, *p* = 0.002, η^2^ = 0.048 (L > M > H), and Total Problems, *F* = 6.16, *df* = 2, *p* = 0.002, η^2^ = 0.048 (L > M > H).

For *DSM* scales, personal strengths categories had significant effect on Anxiety Problems, *F* = 24.99, *df* = 2, *p* = 0.001, η^2^ = 0.171 (L > M > H), and Obsessive-Compulsive Problems, *F* = 6.13, *df* = 2, *p* = 0.003, η^2^ = 0.048 (L > M > H).

The same results were found for female participants. For eight empirical scales including the three broad scales, personal strengths had significant effects except on Thought Problems. In DSM scales, only Somatic Complaints, Inattention, and Hyperactivity-Impulsivity subscales were not found to be affected by personal strengths. Bonferoni correction showed that significant differences were between the higher personal strengths group and the lower personal strengths group, but not with those who reported moderate strengths. Results showed that more personal strengths were associated with fewer emotional and behavioral problems in both participants.

## Multiple Regression Results

Multiple regression was used to test the prediction of ASR scales by traumatic war experience, smoking, drinking alcohol, and well-being for veterans and the wives of veterans. Using Internalizing Problems, Externalizing Problems, and Total Problems as outcome variables and trauma experience, smoking, drinking alcohol and well-being as predictors, we found that the model significantly predicted Internalizing Problems for male participants (*F*(4, 242) = 14.75, *p* < 0.001, with *R* ^2^ = 0.199). Traumatic exposure (*B*_trauma_ = −0.32, *t*(242) = −5.31, *p* < 0.001), drinking (*B*_alcohol_ = −0.17, *t*(242) = −2.93, *p* < 0.001), and well-being (*B*_well-being_ = −0.23, *t*(242) = −3,79, *p* < 0.001) contributed significantly to the model, but smoking (*B*_smoking_ = −0.04, *t*(242) = −1.79, *p* = 0.41) did not. The multiple regression model for male participants with all four predictors also produced significant effects for Externalizing (*F*(4, 242) = 11.69, *p* < 0.001, with *R* ^2^ = 0.16) and Total Problems (*F*(4, 361) = 27.75, *p* < 0.001, with *R* ^2^ = 0.20). Only smoking did not contribute to the multiple regression model for Total Problems and Externalizing Problems (**Table 5**).

**Table 5:** Multiple regression analysis summaries for traumatic experience, wellbeing, smoking and drinking predicting Internalizing, Externalizing and Total problems.

	Internalizing Problems	B	SEB	β	t	p
**Male**	War experience	-.437	.079	-.322	-5.516	.000
	Wellbeing	-.237	.063	-.222	-3.794	.000
	Do you smoke ?	-.484	.591	-.048	-.820	NS
	Do you drink alchohol ?	-3.687	1.258	-.171	-2.931	.004
**Female**	War experience	-.324	.120	-.234	-2.714	.008
	Wellbeing	-.302	.111	-.236	-2.718	.008
	Do you smoke ?	-1.739	.884	-.166	-1.968	.NS
	Do you drink alchohol ?	-2.437	1.667	-.124	-1.462	NS
	**Externalizing Problems**					
**Male**	War experience	-.311	.061	-.302	-5.074	.000
	Wellbeing	-.151	.048	-.187	-3.126	.002
	Do you smoke ?	-.580	.457	-.076	-1.271	NS
	Do you drink alchohol ?	-2.260	.972	-.139	-2.325	.021
**Female**	War experience	-.189	.081	-.208	-2.334	.021
	Wellbeing	-.105	.075	-.125	-1.398	NS
	Do you smoke ?	-1.453	.599	-.211	-2.427	.017
	Do you drink alchohol ?	-1.632	1.129	-.127	-1.446	NS
	**Total Problems**					
**Male**	War experience	-1.182	.212	-.323	-5.569	.000
	Wellbeing	-.642	.167	-.223	-3.838	.000
	Do you smoke ?	-1.356	1.581	-.050	-.857	NS
	Do you drink alchohol ?	-10.537	3.365	-.182	-3.131	.002
**Female**	War experience	-.765	.301	-.221	-2.538	.012
	Wellbeing	-.631	.280	-.197	-2.253	.026
	Do you smoke ?	-4.715	2.228	-.180	-2.116	.036
	Do you drink alchohol ?	-7.072	4.203	-.144	-1.683	NS

For female participants, we found that the model significantly predicted Internalizing Problems (*F*(4, 122) = 8.11, *p* < 0.001, with *R* ^2^ = 0.216). Traumatic exposure (*B*_trauma_ = −0.32, *t*(122) = −2.71, *p* = 0.008) and well-being (*B*_well-being_ = −0.302, *t*(122) = −2.71, *p* = 0.008) contributed significantly to the model, but smoking (*B*_smoking_ = −1.73, *t*(122) = −1.96, *p* = 0.51) and drinking (*B*_alcohol_ = −2.47, *t*(122) = −1.47, *p* = 1.47) did not. The model produced significant effects for Externalizing (*F*(4, 122) = 5.73, *p* < 0.001, with *R* ^2^ = 0.16) in female participants when smoking (*B*_smoking_ = −1.45, *t*(122) = −2.42, *p* = 0.01) was a significant predictor. The model significantly predicted Total Problems (*F*(4, 122) = 7.30, *p* < 0.001, with *R* ^2^ = 0.21), and only drinking did not contribute significantly to the model.

We looked for interaction effects between these four variables for Internalizing Problems, Externalizing Problems and Total Problems. There was a significant interaction effect between war exposure and smoking with Internalizing Problems *F*(4) = 4.31, *p* = 0.002, for veterans, but this was not the case for wives of veterans. Veterans with high exposure during the war and were smokers had more Internalizing Problems than those having low exposure and nonsmokers. We observed an interaction effect between well-being and smoking *F*(6) = 2.86, *p* = 0.012, with Internalizing Problems for veterans. Veterans with poor wellbeing and smokers had more Internalizing Problems compare to those that had better wellbeing and nonsmokers. For the wives of veterans, the interaction effect was not significant for all the scales.

## Discussion

Our multiple regression analysis indicated that traumatic exposure, drinking, smoking, and well-being combined in one model explained 18.5% of Internalizing Problems, 16.4% of Externalizing Problems, and 20.5% of Total Problems for veterans. For the wives of veterans, the model explained 21.6% of the variance in Internalizing Problems, 16.3% of Externalizing Problems, and 19.9% of Total Problems. Results showed that traumatic exposure, drinking, and well-being were most significant predictors for emotional and behavioral problem in veterans and the wives of veterans.

The overall prevalence rates of elevated Internalizing (10.1% in males vs. 11.1% in females), Externalizing (6.9% vs. 7.1%), and Total Problems (8.1% vs. 11.9%) in our study were not high, given that we used a 16th percentile cutoff point using Kosovar norms. In addition, we did not find any effect of gender on prevalence. Our rates are also comparable to those found in other countries, such as in a UK military sample (27), which found a prevalence of 4.5% for any anxious/depressed syndrome, 1.8% for somatization disorder, 18% for alcohol abuse, and 11% for any depressive syndrome.

Other studies that did not use the same questionnaire or methodology have reported that depression was more common than PTSD in war veterans, and with regard to long-term effects, PTSD seems not to be the most common challenge for those who are unwell (28–32). Instead, alcohol, depression, and anxiety disorders are the most commonly observed difficulties in these studies. There are studies that found a high correlation between PTSD and depression (33, 34), suggesting that there may be a substantive relationship between them.

Our finding of few significant gender differences between veterans and wives of veterans is consistent with another study (35) of the prevalence of mental health problems in veterans’ wives seeking help in primary care, which found that they have the same prevalence of problems as their spouses, despite the fact that wives of veterans were less preoccupied with the stigma against receiving primary care services. A study of Vietnamese refugees resettled in Australia for 11 years (36) suggested that they may show good mental health adaptation. Furthermore, the overall service burden of mental disorders was lower for Vietnamese resettled refugees compared with Australian-born respondents. This could reflect cultural differences in the expression of mental distress (37, 38). It could also mean, however, that there was a lack of specialized facilities and services that would allow the veterans and their wives in our study to explore their trauma, which might cause them to turn inward and keep it inside the family system.

Also, the study found that both veterans and wives of veterans had no differences on seeking professional help for their emotional and behavioral problems. Only one veteran and none of the wives of the veterans had asked for professional help, even though 35 veterans and 35 wives of veterans had “Total Problems” scores in the clinical range. Emotional and behavioral distress related to the negative interpersonal effects of the veterans’ and wives of veterans’ untreated invisible wounds may influence the access to services as well. A high percentage of soldiers were not accessing health and psychological care or not receiving adequate treatment (39).

Posttraumatic stress disorder has been the focus of many studies, whereas emotional and behavioral problems more generally do not seem to have been widely addressed. However, some studies have been interested in exploring the connection of emotional problems with PTSD (40). High levels of anxiety were found in veterans with PTSD and in those without PTSD. This leads to the interesting conclusion that high levels of anxiety in disabled veterans might result from still unprocessed traumatic exposure and unsuccessful adaptation to their physical disabilities, but this does not explain why high prevalences have been found in those without PTSD (41). Meanwhile, other studies (42) found that the prevalence of psychiatric disorders was similar in disabled non-Gulf veterans and disabled Gulf veterans (19% vs. 24%).

It was reported that although the symptoms of PTSD and panic did not change after the soldiers returned, the symptoms of depression, anxiety, and alcohol use had increased significantly (43). We may speculate that because of lack of services and high level of stigma, many wives and veterans may remain untreated, even though the changes in family dynamics after the return of veterans from the war and the culture of communication within family make it necessary for all of them. “Veterans we worked with in Kosova may have been forced to cultivate a stoic sense of heroism and devotion to the homeland, but many of them are not able to convey what they really feel concerning the reality: the goal of protecting their family and in particular their children. Many veterans’ families remained without institutional support. They ensured the survival of their family with great difficulty. Our assumption is that they developed an unspoken language that protected them from social pressure but at the same time helped make a distinction between reality and their interior world” (44).

The importance of social support from family and friends is found to be a significant predictor for traumatic symptoms among people who have missing family member(s) as a result of war and who have experienced ambiguous loss (45). Social support was associated with lower levels of both depression and anxiety in a community sample in Mitrovica (46).

It may be speculative, but it seems that the wives of veterans in our sample have managed to synchronize their symptoms within the family even in the absence of specialized services for them and their spouses. This raises the question of whether primary care services are able to provide professional services for the treatment of war-related mental health problems.

A very interesting fact that was not well documented immediately after the end of the war is that a large part of the people in Kosovo who have had health problems, including mental health problems, did not experience exacerbation of their problems in the absence of therapy during the exodus from Kosovo to neighboring countries. Rather, they reported in clinical settings that they were stabilized and that the symptoms returned as soon as they returned home. Mental health resarchers have been attempting to discover the mechanisms that have caused this phenomenon.

Our study found a low prevalence of regular drinking, but the occasional use of alcohol was more common among veterans and wives of veterans. Use of acohol has been reported to be more common among veterans (47–49), but this was not the case in our sample, or at least it is not greater than in other countries, as we had no previous national data for comparison. Our findings are consistent with some similar studies (50), even if they did not use mostly Muslim participants. However, in interpreting these data, we must consider the culture of Kosovo. Over the past 20 years, alcohol and tobacco use has not been a cutural norm in Kosovar families.

Global social-political changes have created far more roles and responsibilities for women. This is true in Kosovo as well. Inadequate preparation of the society for the complex process of female emancipation may correspond to the phenomenon of alcoholism. What is apparent in our society in recent years is the promotion of alcohol to women, which has diminished the proportion of male drinkers. Alcohol is turning into a trend for young people. However, the use of alcohol, in the veteran as well as in the nonveteran population, is not high in our country. Excessive use of alcohol is associated with feelings of insecurity, loneliness, decreased self-esteem, stress, frustrations, discomfort in couple relationships, divided life (partner or part of the family in migration), and health problems (51). An interesting argument might be that the use of alcohol could be a way of dealing with adjustment disorder (52).

The alcohol consumption among military spouses or partners is related to similar factors as among women in the general population (53). Although the percentage of alcohol use has been very low in women, its regular use has been found to be associated with more emotional problems such as anxiety, attention problems, and aggressive behavior. The fact that among the wives of veterans alcohol consumption was associated with behavioral problems is no cause for surprise. While smoking had no significant effects on scales of ASR for veterans, it had a significant effect on veterans’ wives scores in the Internalizing, Externalizing, and Total Problems scales and also in all scales of ASR compared with those in the nonsmoking group. There are studies that support the relationship between alcohol use and smoking and mental health problems (54, 55).

Our study produced interesting results about the influence of sociodemographic variables, such as education, income, and place of living, on emotional and behavioral problems. Wives of veterans living in rural areas showed higher scores on almost all scales of ASR compared with those living in urban areas and to veterans from both urban and rural areas.

Veterans from the low education group had the highest scores compared with other groups. For wives of veterans, there were no differences between groups in all three broad scales of ASR according to education level. Low income was found to be a significant predictor for emotional and behavior problems. Such results are found in other countries too.

All subscales of the ASR were found to have significant mean differences according to traumatic exposure except Intrusive scale for male participants. In all these eight scales, the highest scores came from the higher-exposure group of veterans. Female participants showed significant differences between exposure groups only for Somatic Complaints. The moderate group contains higher scores than the two other groups.

Complex mental health symptoms experienced by people who are faced with traumatic events, such as war, migration, and other types of traumas, place mental health scientists in a position where they must attempt to elucidate the structure of the relationship between symptoms and events. It is not enough to build programs or services that are shaped by explanations that are truncated and not comprehensive or scientific. There are suggestions for providing components for building a verifiable conceptual framework that allows for the understanding of individuals’ mental health, resilience, and adjustment to migration challenges (56), which may be applied to the war experience challenges too.

## Limitations

The findings of this study should be viewed in the context of some limitations. First, we recruited participants from the IOM list of 24,577 war veterans provided by the Kosovar National Association of War Veterans, which was compiled immediately after the war. We used this list because it appeared to be the most reliable source for actual KLA veterans, as opposed to also including people who played a support role in the war but were not fighters, such as supply and medical personnel. The noncombatants who subsequently registered as war veterans have, in fact, become a troubling political issue because they are so numerous and because there is little consensus about whether they should receive the same kinds of benefits as combatants receive. In any event, had we used one of the other longer lists, we might have ended up with a somewhat different sample, including men who were noncombatants, which would have detracted from our study. Second, self-report questionnaires were filled out by the veterans and veterans’ wives, but our participants were not dyads (couples) because of the fact that they were initially recruited to be informants about their children. Third, our participants may not have accurately reported their trauma level. Fourth, the number of drinkers among the participants in each of the groups was very small. We found several interesting interactions, which might be fruitful to explore in observational studies. Finally, because these data were cross-sectional, any proposed causal pathways must be considered with care.

## Conclusion

Parsing out the relationship between the level of exposure to traumatic events and emotional problems and behavior in veterans and their wives would help global mental health researchers address the contextual and experiential dimensions of this relationship. Risk of indirect traumatization including transgenerational transmission underlines the importance of such research. When provided with information on the moderated effects of demographic variables, mental health professionals will be able to identify measurable factors that determine resilience or vulnerability and to better develop and evaluate targeted prevention, treatment, and recovery strategies for mental health problems. Significant training needs to exist among primary care services to improve knowledge of and expertise on veterans’ mental health issues and those of their family members.

## Data Availability

The datasets generated for this study are available on request to the corresponding author.

## Ethics Statement

The study was performed in accordance with the Declaration of Helsinki and was approved by the Ethical Review Committee of the Medical University of Pristina. Written informed consent has been obtained from all study participants.

## Author Contributions

MIS wrote a first draft of the manuscript. LR, MES and SU reviewed and commented the version. MES and LR revised the commented version, which was reviewed again by all coauthors.

## Conflict of Interest Statement

The authors declare that the research was conducted in the absence of any commercial or financial relationships that could be construed as a potential conflict of interest.
